# Prescribing Physical Activity in Parks to Improve Health and Wellbeing: Protocol of the Park Prescription Randomized Controlled Trial

**DOI:** 10.3390/ijerph15061154

**Published:** 2018-06-01

**Authors:** Falk Müller-Riemenschneider, Nick Petrunoff, Angelia Sia, Anbumalar Ramiah, Alwyn Ng, Jane Han, Michael Wong, Tai Bee Choo, Léonie Uijtdewilligen

**Affiliations:** 1 Saw Swee Hock School of Public Health, National University of Singapore, Block MD1, 12 Science Drive 2, #10-01, Singapore 117549, Singapore; nickpetrunoff@nus.edu.sg (N.P.); alwynng2000@yahoo.com.sg (A.N.); ephtbc@nus.edu.sg (T.B.C.); leonie.uijtdewilligen@gmail.com (L.U.); 2 Institute for Social Medicine, Epidemiology and Health Economics, Charite University Medical Centre Berlin, Luisenstrasse 57, 10117 Berlin, Germany; 3 Centre for Urban Greenery & Ecology, National Parks Board Singapore, 1E Cluny Rd., Singapore Botanic Gardens, Singapore 259569, Singapore; ANGELIA_SIA@nparks.gov.sg; 4 Health for Life Centre, Khoo Teck Puat Hospital, Alexandra Health Pte Ltd. 90 Yishun Central, Singapore 768828, Singapore; Anbumalar21@gmail.com (A.R.); han.jane.sy@ktph.com.sg (J.H.); wong.michael.tk@ktph.com.sg (M.W.)

**Keywords:** middle-aged, park, urban green space, physical activity, randomized controlled trial

## Abstract

Previous studies in primary care settings showed that brief advice prescribing physical activity for inactive patients could be an effective way to promote physical activity. Park prescription interventions confer health benefits associated with exposure to nature and increased physical activity by recommending park use specifically to increase physical activity in parks. The purpose of this trial is to evaluate the effectiveness of a park prescription intervention for increasing time spent in moderate-to-vigorous physical activity (MVPA) assessed by accelerometry. Middle-aged Singaporeans who were insufficiently active and who met health screening criteria were recruited via existing community health screening programs and allocated to one of two groups. Intervention participants received a prescription of physical activity in parks, an information pack, access to a weekly group exercise program in parks and telephone counselling (*n* = 80). Control participants received physical activity materials (*n* = 80). The primary outcome (mean difference between both groups in time spent in MVPA minutes per week measured by accelerometer) will be assessed at six months. Secondary outcomes include self-reported health behaviors, self-reported mental wellbeing and objectively-measured physical health. This is the first randomized controlled trial investigating the effectiveness of a park prescription intervention for increasing health-enhancing MVPA.

## 1. Introduction

Physical inactivity is a major contributing factor to increases in non-communicable diseases globally [1]. In Asia, non-communicable disease prevention is a priority for governments to act upon [2]. The Singapore government has sustained efforts to prevent non-communicable diseases and recently declared diabetes prevention a national priority [3,4,5,6].

The prevalence of adequate physical activity engagement according to international guidelines is 76.7% globally and 85.2% in South-East Asia [7]. The population-based 2012 Singapore Health study found that 73.8% of all adults and 70% of adults aged 40–59 years performed sufficient total physical activity [8]. Yet, the same Singaporean study and a separate Taiwanese study found that middle-aged people living in these countries exercise the least [8,9]. Community health screening programs for residents over the age of 40 are freely available throughout Singapore. Results of these screenings have showed that a large proportion of this population is not sufficiently physically active. Therefore, these community health screenings present an opportunity to promote physical activity to this portion of the Singaporean population. Reviews have identified evidence of health benefits associated with exposure to parks and other green space including improved mental health, reduced prevalence of type II diabetes and reduced mortality [10,11,12]. Since most of the available research is from cross-section studies which only provide evidence of associations, it is not possible to infer causal relationships between exposure to parks and health outcomes. A systematic review of the impact of interventions to promote physical activity in urban green space concluded that they appear promising but ‘robust evaluations of such interventions are urgently required’ [13]. Consequently, a need exists to implement stronger studies which support causal arguments for exposure to parks improving health.

The Park Prescription concept emerged from collaboration between the U.S. Centers for Disease Control and Prevention and the National Recreation and Parks Association. In 2013 ‘Park Prescriptions’ were defined as ‘Programs designed in collaboration with healthcare providers … to utilize parks, trails and open space for improving … community health’ [14]. This is closely related to the concept of exercise prescription for people who are not sufficiently physically active [15]. Previous studies which examined prescription of physical activity in primary care settings showed that brief written or oral advice prescribing frequency, intensity, time and type of physical activity for inactive patients could increase physical activity levels [16,17].

Singapore offers an opportunity to explore the application of Park Prescription since there are many accessible and well-managed parks on the relatively small island [3]. Whilst there have been recent studies of prescribing physical activity in parks [18,19], to our knowledge this is the first randomized controlled trial (RCT) to examine the effectiveness.

### 1.1. Study Objectives

The primary objective was to investigate the effectiveness of a park prescription intervention to increase time spent in moderate-to-vigorous physical activity (MVPA) assessed by accelerometry. Secondary objectives included the investigation of intervention effectiveness for:

- improving participants’ total volume of physical activity per week, reflecting average acceleration peroin gravitational units (mg); the amount of light physical activity in minutes per week, the amount of sedentary behavior in hours per week (all assessed via accelerometer);
- improving participants’ self-reported health behaviors including time spent in parks, physical activity time in parks, recreational physical activity and sedentary behavior;
- promoting participants’ self-reported mental well-being; and,
- improving participants’ objectively measured physical health.

## 2. Materials and Methods

The Standard Protocol Items: Recommendations for Intervention Trials (SPIRIT) guidance document for protocols of clinical trials has been used to ensure the content provided in this protocol covers all information that supports the quality of the trial [20].

### 2.1. Study Design

The Park Prescription Trial (PPT) was a parallel group, two-arm, prospective, superiority RCTwith 1:1 allocation ratio to either intervention or control arm. Participants in both arms completed assessments at baseline (V0), 3-month mid-intervention follow-up (V1) and 6-month follow-up at completion of the intervention (V2).

### 2.2. Trial Setting

The PPT was conducted in the community setting in Singapore. Participants were recruited face-to-face or via phone calls and letters to a list of past screening participants. They were recruited at their local community screening program at Khoo Teck Puat Hospital (KTPH) or other community locations organized by KTPH also in the northern area of Singapore or following a health screening of an existing National University of Singapore (NUS) cohort study if their home postal code was also in Singapore’s north. The group exercise component of the intervention was conducted in three regional parks—Woodlands Town Park East, Admiralty Park and Yishun Park—which are near the areas where participants were recruited from. All regional parks in Singapore are well-maintained and these parks have green, landscaped surroundings and shaded areas for the group exercise sessions.

### 2.3. Eligibility Criteria

Subjects who participated in the study met all of these inclusion criteria:

- Singapore citizen or Permanent Resident;
- aged 40–65 years;
- self-reported weekly exercise of <150 min per week;
- blood pressure of ≤139 mmHG (systolic) over ≤89 mmHG (diastolic);
- fasting glucose level of ≤6.0 mmol/L;
- pass the adapted Physical Activity Readiness Questionnaire (PAR-Q) [21];
- able to write and read in English or Chinese; and,
- provide written informed consent.

Pregnant women and those with physical disabilities or lower limb disorders were excluded from participation.

The PAR-Q is a self-guided, nine-question screening tool that can quickly identify conditions or risk factors that require further assessment before engaging in physical activity [21]. For the current trial, we used an adapted version of the PAR-Q (referred to as PAR-Q2), which also included an additional section to formally assess subjects’ age, whether they have physical disabilities or lower limb disorders and the time they spend exercising on a weekly basis.

### 2.4. Intervention

#### 2.4.1. Group 1—Park Prescription Trial (PPT) Intervention

The participants in this group received brief counselling on physical activity along with a park prescription sheet that highlights the importance of engaging in at least 150 min of physical activity per week and the possibility of engaging in physical activity in a park in their neighborhood. The park prescription sheet also includes information on frequency, intensity, time and location of activities and signature fields for the participant and prescriber to sign once they agreed on the prescription. They also received a sheet which was used to plan their weekly physical activity and two information brochures about parks in their neighborhood. Half-way through the trial a trained study team member provided a brief counselling phone call. In addition, they were invited to join in a weekly one-hour outdoor structured and supervised physical activity program in the park for a period of six months. Each one-hour session comprised moderate intensity aerobic activity and strength and balance exercises. The structured physical activity program took place in public parks located in the participants’ neighborhood. The sessions utilized different areas and features of the parks, including walking trails and open spaces, to maximize participants’ exposure to greenery. In the event of inclement weather, the sessions would be held instead at predetermined sheltered indoor locations adjacent to the parks, which had already been booked for that purpose. Participants chose to attend either a session on Tuesday between 7.30–8.30 p.m., or a Sunday morning session mostly between 8.00–9.00 a.m.

The five components of the intervention are described further in Table 1. Protocols were developed to ensure consistent implementation by the study team. All participants were enrolled for approximately six months. Adherence to all components was monitored and recorded by study staff and the proportion that adhered to each component was recorded.

#### 2.4.2. Group 2—Control

Participants in this group continued with their daily routine. They were not given any park prescription or invited to participate in the weekly program in the park. However, they received standard physical activity promotion materials, which were existing publications by the Health Promotion Board, Singapore. In addition, they received all the information materials after Group 1 completed the study and they were also invited to join ongoing exercise classes upon study completion.

### 2.5. Outcomes

Table 2 summarizes the primary and secondary outcomes, the instruments used and the measurement time points.

#### 2.5.1. Primary Outcome

The primary outcome for the study is the mean difference between the intervention group and the control group in time spent in MVPA (minutes per week) objectively quantified via an accelerometer (ActiGraph wGT3X-BT) at the six-month follow-up. This outcome was chosen since it is accepted as an objective physical activity measure that is valid, reliable and practical for use in community trials.

#### 2.5.2. Secondary Outcomes

Secondary outcome measures are defined as the differences between the mean values in the intervention and the control groups at six months follow-up in health behaviors, mental wellbeing and physical health.

Measures for health behaviors related to physical activity include the total volume of activity reflecting average acceleration in gravitational units (mg), time spent in light intensity physical activity and sedentary activity measured by accelerometer, as well as recreational activity time per week, subjectively-measured total MVPA time per week and sedentary time per week assessed by self-administered questionnaire. The physical activity questions come from the previously validated WHO Global Physical Activity Questionnaire [22]. The questions on sedentary time come from the validated International Physical Activity Questionnaire [23]. Self-reported time spent in parks in the past month and time spent doing physical activity in parks in the past month were also included in the self-administered questionnaire at baseline, three months and six months follow-up.

The secondary outcome measures for mental wellbeing include the difference in self-reported quality of life and mental wellbeing between the intervention group and the control group at six months follow-up. This was measured by previously validated survey questions including the general health item from the SF-12, K-10, WHO5 and the WHOQOL-BREF [24,25,26,27]. The physical health measures are objectively-measured Body Mass Index (BMI), fasting blood glucose levels as well as systolic and diastolic blood pressure, as assessed prior to baseline and during the health screening visit at six months follow-up.

### 2.6. Sample Size Calculation

The sample size was estimated based on the primary endpoint of MVPA time per week. After the program, a mean difference in MVPA of 30 (SD = 60) minutes per week between the intervention and the control groups was expected based on existing evidence from physical activity intervention studies (alpha = 0.05 and 80% power) [30]. To detect this effect, a sample of *n* = 64 per group is needed. Assuming a drop-out rate of 20%, this yields a sample size of *n* = 80 per group and *n* = 160 participants in total.

### 2.7. Recruitment

**Figure 1:**
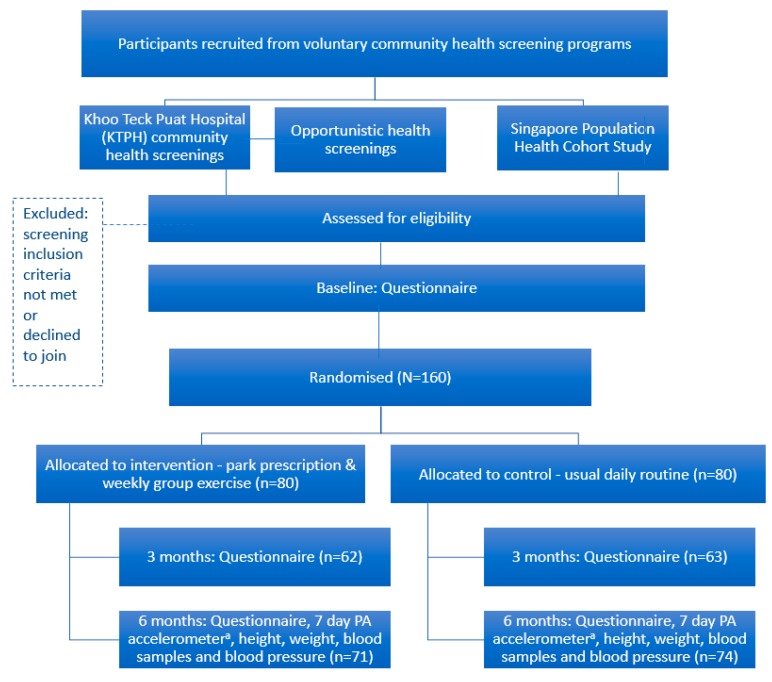
Flow of participants. ^a^ Physical Activity (PA) accelerometer refers to the instrument used to measure the main outcome measure.

Participants who reside in the northern part of Singapore were recruited from KTPH and NUS by trained members of the study team at the respective sites and via letters plus phone calls to a list of past screening participants. Recruitment occurred between April and December 2016. Although the study used additional recruitment mechanisms, approximately 90% of the participants were recruited from the KTPH Community Screening program which is described in detail below. Only 6 participants were recruited by calling 154 participants in an NUS cohort from a list of 307 and an additional 9 were recruited via opportunistic screening mechanisms (e.g., at ad-hoc events). The participant flow is shown in Figure 1.

To increase overall attendance of the health screenings, the study team, research collaborators and persons authorized by the research team promoted the screenings by mechanisms including hanging up posters and handing out flyers in their divisions/departments but also at public venues such as MRT stations. In addition, flyers were distributed and there were ad hoc events where promotion of the screenings also occurred. The screenings were also advertised on relevant NUS and research collaborator websites and Facebook pages. The participants were reimbursed SGD$20 at baseline and SGD$30 after study completion for their time.

#### 2.7.1. Main Recruitment Mechanism: KTPH Community Screening Program

On community health screening days, participants were directed to the NUS PPT booth upon completing their health screening. The NUS team briefly explained the study, administered the PAR-Q2 and handed out the Participant Information Sheet to those who expressed interest and were deemed safe to participate in physical activity after completing the PAR-Q2. Potential participants were informed that activities at the PPT booth occurred for research purposes and that they were not part of the health screening. They were also informed that completion of the PAR-Q2 was voluntary and that completion of the PAR-Q2 did not mean they had to participate in the research. A unique study ID was noted on each PAR-Q2 form. Participants’ National Registration Identity Card (NRIC) number was noted on a separate sheet, listed after their respective study ID. A list containing the NRIC numbers of those who passed the PAR-Q2 was handed to the KTPH health screening team. Subsequently, a study nurse checked participants’ blood pressure values and fasting glucose levels obtained from the health screening. Those who met the selection criteria were shortlisted for the study.

During the following days, eligible people received a text message from the health screening team, prompting them to pick-up their report earlier during the report collection day (report collection days were generally about 2 weeks after the health screening day) and informing them of the benefits of the study. Also, eligible individuals received a reminder phone call from NUS or KTPH staff one day prior to the report collection if this was logistically feasible. During this phone call, eligible individuals had the opportunity to ask questions about the content of the study. During the report collection day, a colored sticker was placed on the report envelope of people who were assessed as eligible by the health screening team. Eligible people were then directed to the PPT booth after collecting their reports and attending the subsequent health talks. The NUS study team then explained the study in detail, took their written informed consent and enrolled them into the study.

### 2.8. Participant Timeline

Figure 1 illustrates the participant flow and includes the time schedule of enrolment, intervention components, assessments and visits for participants.

### 2.9. Assignment of Interventions

Participants were randomized into one of the two groups based on computer-generated random numbers using Stata statistical software version 12 [31]. Block sizes were generated randomly using a minimum block size of four and a maximum block size of ten. The group assignments were handed to a separate study team member to be placed into sequentially-numbered opaque envelopes and sealed. The envelopes had the participant ID documented on the outside according to the randomization lists and a slip of paper stating ‘intervention’ or ‘control’ was placed in each envelope. The envelopes were opened by the study team member who would perform the park prescription counselling component of the intervention after confirmation of eligibility, provision of informed consent and completion of the baseline assessment in the presence of the study participants, which ensured allocation concealment. Due to the nature of the intervention and the logistics of the study, participants and research staff were subsequently not blinded to the group allocation. 

### 2.10. Data Collection and Analysis

#### 2.10.1. Data Collection

Table 2 summarizes data collection measures and instruments at each of the three measurement time points. The types of measures are also described in detail under Section 2.5 Outcomes and this section provides information on the procedures used to ensure quality of data collection and completeness of follow-up.

At baseline, secondary outcome measures of physical health including blood chemistry (blood lipids, fasting blood glucose), blood pressure (systolic and diastolic) and anthropometry (height and weight) had been collected prior to enrolment in the trial as part of existing health screenings or as part of data collection for the existing NUS cohort study. The data at these health screenings are collected by nurses who are trained in standard procedures for collecting the anthropometric data accurately and reliably and taking blood safely. The questionnaire, which combined items from several validated questionnaires, was piloted with a small sample who were of the same age range as study participants. Pilot participants were from the National University Hospital clinics. The pilot was used to see that the time to completion was acceptable and all questions were understood. Staff were also instructed to check for missing data and plausibility. To increase participant retention, participants’ contact details were collected at baseline for follow-up.

At the three-month follow-up, self-administered questionnaires were mailed to participants for completion with a stamped, addressed return envelope as well as a letter thanking them for their participation and encouraging them to return the questionnaire within one week. If the surveys were not returned, participants received reminder text messages and if necessary follow-up phone calls. Participants who did not complete the survey at three months were still followed up at six months.

At six-month follow-up only, participants received a letter which invited them to attend a follow-up health screening at KTPH. In most cases the letter, including the questionnaire, accelerometer and instructions for wearing it were hand delivered to participants near the end of the program at their homes so an explanation on wearing the accelerometer could be provided. The accelerometer was worn on the participant’s wrist for seven days. The instructions explained that it must be worn on the wrist of the non-dominant hand at all times for seven consecutive days as soon as possible after receiving it. To increase the chances of collecting complete data for at least four valid days for each participant, a trained study team member collected the accelerometer and downloaded the data to a laptop with the full ActiLife software installed. The study team member checked the number of complete days of data provided and if there were less than four they asked the participant to wear another accelerometer for another seven days.

During the scheduled visit at KTPH, participants answered the questionnaire (if it had not been completed in advance), returned the accelerometer and underwent a health screening. For participants in the intervention arm, the questionnaire also assessed satisfaction with the phone counselling component of the program as well as the program overall. To maximize attendance at the six-month follow-up health screening session participants were reminded that they would receive their final $30 cash incentive at this session.

#### 2.10.2. Data Management

Hard copies of the baseline and follow-up questionnaires are stored in a locked cabinet in the Principal Investigator’s (PI’s) office at the Saw Swee Hock School of Public Health (SSHSPH), NUS. All participants were given a unique identification number. This identification number was used on all measurements collected. These were only accessible to members of the research team who needed to access them. All data were de-identified using the unique identification number. 

The data from the questionnaires will be entered into a spreadsheet by trained research staff. This data will only be accessible to members of the research team who require access to it. All datasets will be de-identified and no names or contact information will be part of the datasets. A master key, or ‘participant identity log’ will only include participant names, contact information and identification numbers. This master key, or ‘participant identity log’ will be kept separately from the main research data in a password-protected document. Datasets will be stored on a secure centralized computer within SSHSPH, which is password-protected. Additional backup files will be on external hard drive disks. All data sets will be under password protection. All team members agree to maintain in strict confidence the names, characteristics, questionnaire scores, ratings, incidental comments, and/or other information on all subjects and/or subjects’ data they encounter. The PI will have access to the centralized computer that contains the research data. Study team members will have access to the data on their computers (which are password-protected). Research data used in publication will be kept for a minimum of 10 years before being discarded. All research-related data will be erased and mediums will be disposed of in a way that ensures that research data cannot be retrieved.

#### 2.10.3. Statistical Analysis

For demographic variables of the intervention and control groups at baseline, descriptive statistics will be presented using mean and standard deviation for variables that are normally distributed and median and interquartile range for skewed continuous variables and proportion for categorical variables. Table 3 provides an overview of all primary and secondary outcome measures, their definitions and the methods to be used for evaluating the respective hypotheses.

For the continuous primary outcome of MVPA as well as continuous secondary outcomes based on health behavior, mental health and physical health measures at the six-month follow-up, the mean difference between the intervention and the control groups will first be evaluated using t-tests. Further adjustment for respective baseline values of the outcome variables (where available) of the individual will be made via multiple linear regression analysis. For accelerometer related outcomes where no baseline value is available, adjustment for total self-reported MVPA of the individual will be made via multiple linear regression analysis. For categorical outcomes at 6-months (e.g., depression/anxiety levels from K10) logistic regression will be conducted adjusting for the same covariates.

Subgroup analysis based on the report of engagement in physical activity in parks will be performed to investigate the effect of the intervention on the primary and secondary outcomes.

Up-to-date versions of R (Auckland, New Zealand) and STATA (Texas, TX, USA) will be used to conduct the analysis. All evaluations will be made based on intention to treat, assuming a two-sided test at the 5% level of significance. Effect sizes and 95% confidence intervals will also be reported for the respective outcome measures.

### 2.11. Ethics and Dissemination

#### 2.11.1. Research Ethics Approval

This trial and all its associated forms and resources, have been approved by the National Healthcare Group Domain Specific Review Board (DSRB) in Singapore (2015/00611-Park Prescription Trial).

#### 2.11.2. Declaration of Interests

One investigator is a representative of the National Parks Board (NParks) in Singapore. NParks provided the funding for the project. To ensure no influence on the outcomes of the study, NParks representatives were only involved in the conceptualization of the study and had some input into design. All other aspects of the study were conducted independently by staff from NUS and KTPH.

#### 2.11.3. Dissemination Policy

The research data will be compiled into a poster that may be used for conferences (both local and overseas). The research data will also be compiled and be written into a paper that will be sent for publishing in a peer-reviewed journal. In addition, we intend to share the key findings on the NParks website and on other relevant public forums and/or (inter)national newsletters. The content and type of media will always be agreed on among the organizations collaborating to conduct the trial.

## 4. Discussion

There is increasing interest in the health benefits of parks and other urban green spaces since epidemiological evidence suggests that exposure to green spaces in urban environments is associated with physical and mental health benefits [10,11,12,32]. A systematic review of the impact of interventions to promote physical activity in urban green space identified 12 studies with a control comparison and only one of these was an RCT [13]. Whilst seven of the twelve studies achieved positive impacts on physical activity and park usage, the single RCT the review identified was the only study that was assessed as being at low risk of bias [33].

The Park Prescription Trial is prospective, includes a control group, is randomized, incorporates an objective physical activity measure and is hypothesized to increase the utilization of urban green spaces. This RCT will evaluate the effectiveness of the Park Prescription intervention for increasing health-enhancing moderate-to-vigorous physical activity. It will also assess impacts on health behaviors including park use, whilst incorporating an assessment of mental wellbeing and physical health. Since there are no known RCTs of this novel approach to promoting health and well-being by prescribing physical activity in parks, this trial will make a significant contribution to physical activity intervention research and research into the health benefits of urban green spaces.

This study has several strengths, including a sample size that has been calculated to ensure adequate statistical power to detect the main outcome measure. Objective and valid data collection methods have been used for most of the outcome measures. A six-month follow-up will determine if the changes made during the intervention can be maintained. Since it is an effectiveness trial conducted in a community setting, it is likely to be more reproducible than an intensively-resourced efficacy study, which in turn increases the chances of the intervention being appropriate for scaling up to benefit larger segments of the population [34,35]. To aid this process of potential translation of this research for broader adoption there is also a complementary process evaluation which will explore implementation issues (reach, adoption, fidelity, satisfaction) as well as assess which aspects of the program may contribute to potential impacts (i.e., why the program achieved the results it achieved) using both quantitative and qualitative methods. Finally, the development of the intervention involved formative research and elements of this were informed by the PRECEDE-PROCEED theoretical model [36].

There are some risks and limitations associated with the trial. Community trials tend to be susceptible to loss to follow-up amongst participants. For this reason, intensive follow-up strategies were implemented and intention-to-treat analysis will be performed to reduce potential biases this may introduce. An intervention of this duration and intensity may not be expected to achieve large impacts on the chosen physical health indicators which include objectively-measured anthropometric, blood biochemical and blood pressure measures. However, these measures were part of existing health screenings that participants already had at baseline so there was an opportunity to assess potential impacts with only one additional measurement point.

## 5. Conclusions

Park Prescription is a novel approach to promoting participation in physical activity and exposure to urban green space. Therefore, a rigorous approach to evaluation of the Park Prescription Intervention was warranted. If found to be effective, health systems could translate the Park Prescription concept into broader use in primary care settings since it is relatively simple to implement. In the Singapore context, this intervention could benefit a large segment of the population if it were linked with existing screening programs which are offered free to all Singaporeans over 40 years of age.

## Figures and Tables

**Table 1:** Intervention components and intervention delivery timing.

Component	Description	Delivery Timing
Counselling	Face-to-face physical activity and park use counselling.	Commencement
Park Prescription	Participants completed a sheet with a trained study team member. The prescription section of the participant’s sheet outlined a goal they committed to which specified the frequency, intensity, time and location of exercise in parks. The study team retained a separate sheet with an assessment of the participant’s baseline activity level—low, moderate or active.	Commencement
Materials	Participants received two brochures: one developed for the Trial provided information on the main parks in the northern part of Singapore and their features, including walking trails (with time needed to complete them) and fitness corners. The other was a general brochure from the Singapore National Parks Board containing a map and information on the Northern Explorer Loop (a series of parks in Singapore’s north connected by a network of walking and cycling paths). A planning sheet, where participants filled in the types of activities they aimed to do each week over the trial period, also included information and examples.	Commencement
Follow-up counselling	Brief phone call counselling by a trained study team member. The counselling assessed progress towards set goals and included modification of those goals if necessary.	Three months
Group exercise	Structured exercise program delivered in parks by a trained group exercise instructor. To encourage attendance, participants received mobile text message reminders prior to each weekly exercise session.	Weekly over six months.

**Table 2:** Outcome measures and instruments for data collection at each time point.

Primary Outcome	Measurement	Instrument	V0	V1	V2
MVPA	Time spent in activity of moderate to vigorous intensity	Accelerometer			X
**Secondary outcome**					
Physical activity	Total physical activity volume ^a^	Accelerometer			X
Light and sedentary activity	Time spent in light and sedentary activity	Accelerometer			X
Physical activity	Frequency, intensity, time and type	Self-administered questionnaire—GPAQ	X	X	X
Sedentary activity	Time spent sitting	Self-administered questionnaire—IPAQ	X	X	X
Park usage	Time spent in parks last month, physical activity time in parks on a typical month.	Self-administered questionnaire	X	X	X
Mental wellbeing	Wellness	Self-administered questionnaire—SF-12, K10, WHO5, WHOQoL-BREF ^b^	X		X
Anthropometry	BMI **^c^**—weight in kilograms divided by height in meters squared	BMI machine	X		X
Biochemistry	Blood lipids, fasting blood glucose	Blood samples	X		X
Blood pressure	Systolic and diastolic	Dinamap blood pressure monitor	X		X
**Process indicator ^d^**					
Motivation to exercise	Social support for physical activity, reasons to engage, physical activity knowledge	Self-administered questionnaire—BREQ-2		X	
Attitudes and behaviors	Perceived changes in participants’ attitudes and behaviors towards physical activity and park use, intention to continue group exercise	Self-administered questionnaire.			X
Satisfaction with and quality of program	Satisfaction with and quality of prescription sheet and parks brochure	Self-administered questionnaire		X	
	Satisfaction with and quality of physical activity planning sheet	Self-administered questionnaire		X	
	Satisfaction with and quality of phone counselling	Self-administered questionnaire			X
	Satisfaction with and quality of program overall	Self-administered questionnaire			X

V0 = Baseline, V1 = 3-month mid-intervention follow-up, V2 = 6-month follow-up at completion of the intervention. GPAQ Global Physical Activity Questionnaire (sections on work, transportation and recreational activities); IPAQ International Physical Activity Questionnaire, Part 5: Time Spent Sitting, item 26 and 27; SF-12, 12 item short-form survey, item 1; K10 Kessler Psychological Distress Scale; WHO5 WHO (Five) Well-being Index; WHOQoL-BREF WHO Quality of Life Short Form; BREQ-2 Exercise Regulation Questionnaire. ^a^ We use Euclidean Norm Minus One (ENMO) as the ‘total volume’ measure. It reflects ‘average acceleration’ in gravitational units (mg). [28,29]. ^b^ WHOQoL-BREF administered only at 6-month follow-up. **^c^ Body Mass Index (BMI). ^d^** Process indicators only administered to intervention group.

**Table 3:** Outcome measures, their definitions and methods for evaluating the hypotheses.

Outcome	Hypothesis	Definition	Analysis
(1) Primary			
Time spent on MVPA ^a^—objective measure	Improvement in MVPA in intervention group as compared to control group at six months	Time spent on MVPA in minutes per week as measured by the accelerometer	*t*-test. Linear regression
(2) Secondary			
a. Health behaviors	Improvement in health behaviors in intervention group as compared to control group at six months		
Total volume of physical activity		Total activity volume as measured by the accelerometer ^b^	*t*-test. Linear regression
Time spent on light and sedentary activity		Time spent per week on light and sedentary physical activity as measured by the accelerometer	*t*-test. Linear regression
Time spent on MVPA—subjective measure		Self-reported time (minutes) per week spent on MVPA as recorded in questionnaire	*t*-test. Linear regression
Time spent in parks; time spent being physically active in parks		Self-reported time (minutes) in parks in the past month; and time spent engaging in physical activity in parks in a typical month as recorded in the questionnaire	*t*-test. Linear regression
Recreational MVPA time		Self-reported time (minutes) per week spent on recreational activity as measured by GPAQ^a^	*t*-test. Linear regression
Sitting time		Self-reported time (minutes) per week spent sitting as measured by IPAQ ^a^	*t*-test. Linear regression
b. Mental wellbeing	Improvement in mental wellbeing in intervention group as compared to control group at six months		
Mental wellbeing		Self-reported mental wellbeing as measured by SF-12 (1-item, general health), K-10, WHO5 and WHOQOL-BREF^a^	*t*-test. Linear regression. Logistic regression
c. Physical health	Improvement in physical health in intervention group as compared to control group at six months		
Body Mass Index (BMI)		Weight in kg divided by height squared in m measured by BMI machine.	*t*-test. Linear regression
Fasting blood glucose		Fasting blood glucose in mmol/L. Laboratory assessment	*t*-test. Linear regression
Systolic and diastolic blood pressure		Systolic and diastolic blood pressure in mmHG measured by a Dinamap blood pressure monitor	*t*-test. Linear regression

^a^ Abbreviations: MVPA, moderate-to-vigorous physical activity; GPAQ, Global Physical Activity Questionnaire; IPAQ, International Physical Activity Questionnaire. SF-12, 12 item short-form survey; K10, Kessler Psychological Distress Scale; WHO5, WHO (Five) Well-being Index; WHOQoL-BREF, WHO Quality of Life Short Form; BREQ-2, Exercise Regulation Questionnaire.^b^ We use Euclidean Norm Minus One (ENMO) as the ‘total volume’ measure. It reflects ‘average acceleration’ in gravitational units (mg) [28,29].

## Supplementary Materials

The informed consent materials and intervention materials are available online at http://www.mdpi.com/1660-4601/15/6/1154/s1.

## Trial Registration

ClinicalTrials.gov Identifier: NCT02615392. Registered 23 November 2015.
