# Supplementary material for: Prescribing Physical Activity in Parks to Improve Health and Wellbeing: Protocol of the Park Prescription Randomized Controlled Trial
Source: Int J Environ Res Public Health. 2018 Jun 1;15(6):1154. doi: 10.3390/ijerph15061154 (PMC6025310; doi:10.3390/ijerph15061154)
Supplement: Supplementary file 1 [file ijerph-15-01154-s001.zip › Park Prescription Trial_PARQ2_Supplementary_file_for_PPT_Protocol_Paper.pdf]

Physical Activity Readiness Questionnaire PAR-Q (Circle on the form)

Eligibility check (Note and circle on the form)

Age (40-65)		
Able to write/read	Yes	No
Lower limb disorders	Yes	No
Physical disabilities	Yes	No
How much do you exercise per week, on most weeks?<br><br>Examples;<br>Moderate- intensity aerobic activity (e.g. brisk walking) or Vigorous- intensity aerobic activity (e.g. running, jogging or playing basketball)?	☐ up to 30 minutes<br>☐ up to 1 hour (less than 60 minutes)<br>☐ up to 1,5 hours (less than 90 minutes)<br>☐ up to 2 hours (less than 120 minutes)<br>☐ up to 2,5 hours (less than 150 minutes)<br>☐ more than 2,5 hours (150 minutes or more)	

**1.** Has anyone in your immediate family (mother, father, sister or brother) had a heart attack or died suddenly of a heart related disorder before age 55 (men) or 65 (women)?	Yes	No
**2.** Has your doctor informed you that you have any of these conditions? (check all that apply)		
☐ Heart condition or disease (also includes any type of heart surgery)	Yes	No
☐ Stroke	Yes	No
**3.** Has your doctor informed you that you have any of these conditions? (check all that apply)		
☐ Lung disease (e.g. chronic obstructive pulmonary disease/COPD or asthma)	Yes	No
☐ Diabetes	Yes	No
**4.** In the past 1 year, have you had chest pain when you engage in physical activity or when at rest?	Yes	No
**5.** Do you ever experience dizziness or even lose consciousness?	Yes	No
**6.** Do you have any bone, joint or muscle problem (e.g. back, knee, hip, shoulder or ankle) that could be made worse by participating in exercise?	Yes	No
**7.** Do you take tablets for high blood pressure and either continue to have **poorly controlled** high blood pressure, or you **do not** follow up with a doctor on a regular basis?	Yes	No
**8.** Are you currently pregnant?	Yes	No
**9.** Do you know of any reason why participating in exercise or any other physical activity might be harmful to your health?	Yes	No

Participant ID:

____

I have answered all previous questions honestly and to the best of my knowledge.
I hereby declare the above to be true reports of my medical history and current conditions and feel fit to participate in physical activity.

Participant Signature: ____

Date: ____

NUS Staff Name: ____

NUS Staff Signature: ____

Date: ____
