# Supplementary material for: Prescribing Physical Activity in Parks to Improve Health and Wellbeing: Protocol of the Park Prescription Randomized Controlled Trial
Source: Int J Environ Res Public Health. 2018 Jun 1;15(6):1154. doi: 10.3390/ijerph15061154 (PMC6025310; doi:10.3390/ijerph15061154)
Supplement: Supplementary file 1 [file ijerph-15-01154-s001.zip › Park_Prescription_Trial_Informed_Consent_Supplementary_file_for_PPT_Protocol_Paper.pdf]

INFORMED CONSENT FORM

1. Study Information

Protocol Title:

Park Prescription Trial

Principal Investigator & Contact Details:

Dr. Falk Müller-Riemenschneider
Saw Swee Hock School of Public Health, National University of Singapore
Tahir Foundation Building, 12 Science Drive 2, #10-01
Phone: (65) 6516 4988 (institutions main line)
Email: falk.mueller-riemenschneider@nuhs.edu.sg

Collaborating Sponsor:

National Parks Board Singapore

2. Purpose of the Research Study

You are invited to participate in a research study. It is important to us that you first take time to read through and understand the information provided in this sheet. Additionally, before you take part in this research study, the study will be explained to you and you will be given the chance to ask questions. After you are properly satisfied that you understand this study, and that you wish to take part in it you are asked to provide your consent.

The main objective of this study is to test the effectiveness of prescribing physical activity in parks (i.e., receiving a ‘park prescription’), in order to improve physical activity levels in Singaporean adults aged 40-65 years.

The study will recruit 160 adults from the Sembawang, Nee Soon and/or Woodlands Divisions who join the Population Health Community Screening Programme. The study team consist of researcher from the National University of Singapore (NUS), employees of the Khoo Teck Puat Hospital (KTPH) and the National Parks Board Singapore.

3. What procedures will be followed in this study

If you take part in this study, you will randomly allocated to one of the two study groups. The two study groups are as follows:

Group 1: The participants in this group will receive a brief counseling on physical activity together with a park prescription that highlights the importance of engaging in at least 150 minutes of physical activity per week and the possibility of engaging in physical activity in the park. In addition, they are invited to join in a structured and supervised physical activity program in the park. The structured physical activity program will take place in public parks located in the participants’ neighbourhod. Participants will receive text messages for reminder and registration purposes approximately once a week. Also, participants will receive a sheet to monitor their weekly physical activity, information about parks in their neighborhood, and a counseling phone call half-way through the study.

Group 2. The participants in this group will not be given any park prescription or be invited to participate in the weekly program in the park. However, they will receive all the information materials provided to Group 1 after the study has ended.

Your participation in the study will last approximately 6 months. If you agree to take part in this study, the following will happen: during report collection, NUS researchers will ask you whether you have read this information letter and are interested in participating in the study. Before deciding on your participation, you will have time to ask the NUS researcher

questions related to the study and the consent. If you decide to participate in the study NUS researcher will ask you to provide the informed consent form with your signature.

The study schedule below gives an overview of the different phases of the study.

Study schedule

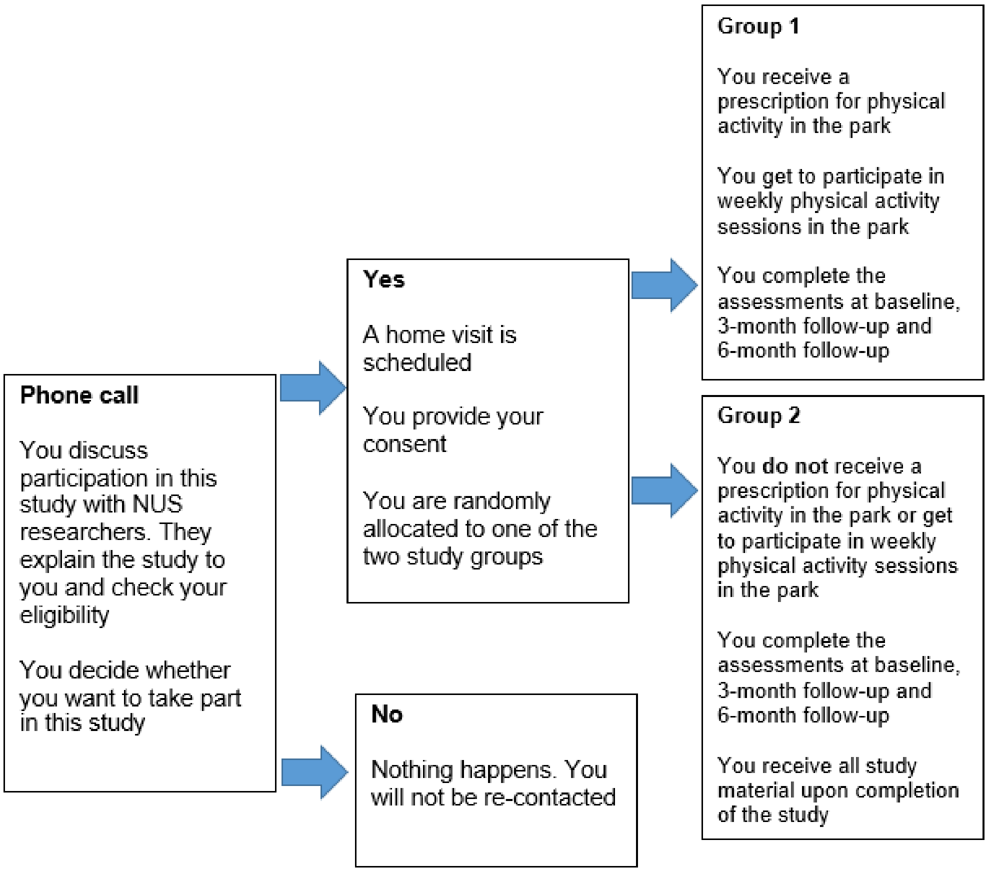

During the course of the study, there will be three assessment points:

1. *Baseline (at the beginning of the study)*: You will be asked to fill in a questionnaire asking you about your health, health behaviours and park usage. Filling in the questionnaire will take you about 30 minutes, and will be done during the report collection right after you have consented to participate in the study.

2. *3-month follow-up*: You will be asked to fill in a questionnaire which is similar to the baseline questionnaire in content and length. The questionnaire will be delivered to you.

3. *6-month follow-up*: You will be asked to fill in a questionnaire which is similar to the baseline questionnaire in content and length. In addition, you will be requested to wear an electronic device on your wrist for 7 consecutive days to measure your physical activity. The questionnaire and device will be delivered to you. Finally, you will be asked to visit KTPH for assessing: 1) your height, weight, waist circumference and blood pressure; and 2) your blood sugar and lipid profile. The latter will be examined by taking a blood sample in the same way as it was done during the health screening. Approximately 1 teaspoon (5ml) will be drawn in one instance.

In order to spare you from additional assessments and save you time, your health data from the screening will be linked with the data collected during this study.

After the study has ended, you may be invited for a group discussion to evaluate the

program. The group discussion will be held at a venue in your neighborhood and will take approximately 1,5 hours. Engaging in the group discussion is optional. You can also participate in the study without taking part in the group discussion.

4. Possible Risks from Participating in the Study

Participating in physical activity can involve some limited risks of injury and detrimental health effects. However, these risks are not greater than during physical activities of daily living. Also, drawing blood may cause slight bruising and bleeding around the site of injection, and there is a limited chance of experiencing pain or fainting. Generally, the risks associated with this study should be very low and are further minimized by appropriate instructions and information provided to participants and the research team. We do not anticipate any health- or other related risks for study participants who attend the group discussions to evaluate the program. The most apparent burden for participants is the time investment.

Research results will be distributed to you after completion of the study.

5. Possible Benefits from Participating in the Study

Participating in this study may increase your physical activity and thereby improve your health and general well-being. Also, your participation makes it possible to test the effectiveness of prescribing physical activity in parks, a strategy which could potentially be applied in future community screening across Singapore.

6. Alternatives to Participation

If you choose not to take part in this study, you will continue to be with the Population Health Community Screening Programme. Not participating in the study will not affect any follow up provided by the screening programme.

7. Costs & Payments if Participating in the Study

Participation in this study will not cost you anything except for time. This study is performed at no charge to you. You will be reimbursed for your time, inconvenience and transportation costs as follows:

- When you have completed the baseline assessment S$20 in cash will be given to you. Another S$30 will be given to you at the 6-month follow-up.
- If you do not complete any assessment for any reason, you will not receive the reimbursement.

8. Voluntary Participation

Your participation in this study is voluntary. You may stop participating in this study at any time. Your decision not to take part in this study or to stop your participation will not affect your participation in the Population Health Community Screening Programme or any benefits to which you are entitled.

If you withdraw from the study, you will be required to call or write to (email/mail) the Principal Investigator. The Principal Investigator will confirm your withdrawal in writing, either by sending you a reply email or letter. Your withdrawal will be registered electronically. However, the data that have been collected until the time of your withdrawal will be kept and analysed. The reason is to enable a complete and comprehensive evaluation of the study.

In the event of any new information becoming available that may be relevant to your

willingness to continue in this study, you will be informed in a timely manner by the Principal Investigator or his/her representative.

9. Confidentiality of Study and Medical Records

Information collected for this study will be kept confidential. Your questionnaire answers, physical activity information coming from the device and health assessments, to the extent of the applicable laws and regulations, will not be made publicly available.

However, the Collaborating Sponsor *(National Parks Board)*, and NHG Domain-Specific Review Board and Ministry of Health will be granted direct access to your original data to check study procedures and data, without making any of your information public.

By signing the Informed Consent Form attached, you (or your legally acceptable representative, if relevant) are authorizing (i) collection, access to, use and storage of your "Personal Data, and (ii) disclosure to authorised service providers and relevant third parties.

"Personal Data" means data about you which makes you identifiable (i) from such data or (ii) from that data and other information which an organisation has or likely to have access. This includes medical conditions, medications, investigations and treatment history.

Research arising in the future, based on this Personal Data, will be subject to review by the relevant institutional review board.

By participating in this research study, you are confirming that you have read, understood and consent to the Personal Data Protection Notification available at http://www.nus.edu.sg/images/resources/content/misc/pdpa.pdf

10. Compensation for Injury

By signing this consent form, you will not waive any of your legal rights or release the parties involved in this study from liability for negligence.

Injuries arising outside the scope of this research study will not held responsible by this research study. However, if you are injured as part of the exercise program, you will be compensated under NUS clinical trial liability insurance policy.

11. Who To Contact if You Have Questions

If you have questions about this study, you may contact Dr. Léonie Uijtdewilligen. She can be reached via email at: leonie_uijtdewilligen@nuhs.edu.sg.Or by phone: (65) 6601 5006. Alternatively, you may contact the Principal Investigator, Dr. Falk Müller-Riemenschneider. He can be reached via email at falk.mueller-riemenschneider@nuhs.edu.sg. Or by phone: (65) 6516 4988.

The study has been reviewed by the NHG Domain Specific Review Board (the central ethics committee) for ethics approval.

If you want an independent opinion to discuss problems and questions, obtain information and offer inputs on your rights as a research subject, you may contact the NHG Domain Specific Review Board Secretariat at 6471-3266. You can also find more information about the NHG Domain Specific Review Board at www.research.nhg.com.sg.

If you have any complaints or feedback about this research study, you may contact the Principal Investigator or the NHG Domain Specific Review Board Secretariat.

CONSENT FORM FOR GROUP DISCUSSION

Protocol Title:
Park Prescription Trial

Principal Investigator & Contact Details:

Dr. Falk Müller-Riemenschneider
Saw Swee Hock School of Public Health, National University of Singapore
Tahir Foundation Building, 12 Science Drive 2, #10-01
Phone: (65) 6516 4988 (institutions main line)
Email: falk.mueller-riemenschneider@nuhs.edu.sg

Please tick the boxes that apply

☐ I voluntarily consent to take part in this research study. I have fully discussed and understood the purpose and procedures of this study. This study has been explained to me in a language that I understand. I have been given enough time to ask any questions that I have about the study, and all my questions have been answered to my satisfaction.

☐ By participating in this research study, I confirm that I have read, understood and consent to the (Institution) Personal Data Protection Notification. I also consent to the use of my Personal Data for the purposes of engaging in related research arising the future.

☐ I agree to be re-contacted for future research related only to this study for a period of at least 6 years after completion of this study.

Name of Participant	Signature	Date

Investigator Statement
I, the undersigned, certify that I explained the study to the participant and to the best of my knowledge the participant signing this informed consent form clearly understands the nature, risks and benefits of his/her participation in the study.

Name of Investigator / Person administering consent	Signature	Date

- PLEASE NOTE DOWN CONTACT DETAILS ON THE BACKSIDE OF THIS SHEET -

Contact Information
For the purpose of:
- delivering the questionnaire and at 3-month follow-up
- phone counseling at 3-month follow-up (only for Group 1)
- sending text messages for the program in the park (only for Group 1)
- delivering the questionnaire and electronic device at 6-month follow up
- contacting for program evaluation

Name:____

Address:____

____

____

Phone:____

Email:____
